# Different treatments for 3- or 4-part proximal humeral fractures in the elderly patients: A Bayesian network meta-analysis of randomized controlled trials

**DOI:** 10.3389/fsurg.2022.978798

**Published:** 2022-09-29

**Authors:** Jiale Guo, Caiju Peng, Ziyan Hu, Yehai Li

**Affiliations:** Department of Orthopedics, Chaohu Hospital of Anhui Medical University, Hefei, China

**Keywords:** proximal humeral fractures, conservative, locking plates, intramedullary nails, hemiarthroplasty, reverse shoulder arthroplasty

## Abstract

**Background:**

Proximal humeral fractures are the third most common fracture in the body, and their incidence is rising year by year as the population ages. However, the treatment of the proximal humerus in parts 3 and 4 is still debatable, necessitating a network meta-analysis to determine the best treatment for each treatment modality.

**Methods:**

We searched PubMed, Embase, Cochrane Library for randomized controlled trials on proximal humeral fractures up to June 21, 2022. We performed data extraction and literature quality assessment by two independent authors and extracted constant score and reoperation rate as indicators for evaluation. Stata software, Revman software, JAGS software and the R-based BlandAltmanLeh package, gemtc package and riags package were used to perform this Bayesian network meta-analysis.

**Results:**

Following screening, 11 papers with a total of 648 participants were included in the analysis. The SUCRA values for the constant score were in the following order: RSA, IMN, Conservative, HA, and LP, and the SUCRA values for the reoperation rate were LP, HA, IMN, Conservative, and RSA.

**Conclusion:**

The elderly with 3- or 4-part proximal humeral fractures should consider RSA because it received the best evaluation ranking in terms of constant score and reoperation rate.

**Systematic Review Registration:**

https://www.crd.york.ac.uk/prospero/display_record.php?ID=CRD42022341209, identifier: CRD42022341209.

## Introduction

For the elderly, proximal humerus fracture (PHF) is the third most frequent fracture after hip fracture and distal radius fracture (1). Proximal humerus fractures account for about 5%–6% of all fractures in the body (2), and it is one of the four major osteoporotic fractures in the human body (3, 4) (hip fractures, vertebral compression fracture, distal radius fracture, and proximal humerus fracture), with its main factors being falls and osteoporosis. Most proximal humeral fractures can be treated conservatively (5), with results comparable to surgical treatment (6), but the treatment of displaced three- or four-segment fractures is currently debatable. Currently, for displaced three- or four-part proximal humerus fractures, the treatment options include: non-surgical conservative treatment (Conservative), internal fixation with locking plates (LP), internal fixation with intramedullary nails (IMN), hemiarthroplasty (HA), and reverse shoulder arthroplasty (RSA). As the population ages, the incidence of proximal humeral fractures is increasing, and an epidemiological study predicts that the incidence of PHF could increase to three times the current rate in the next three decades (7). The increase in the incidence of PHF has been accompanied by changes in the use of various treatment measures. In Australia, the incidence of PHF increased from 26.8 per 100,000 person-years in 2008 to 45.7 per 100,000 person-years in 2017 (8). However, the proportion of PHF treated surgically dropped from 32.5% to 22.8%; open reduction internal fixation (ORIF) use dropped from 76.6% to 72.6%; HA use significantly decreased from 19.3% to 3%; and RSA use significantly increased from 4.1% to 24.5%. Similar trends were observed in the US, where the proportion of PHF treated surgically decreased from 16.2% to 13.9% between 2009 and 2012; ORIF use did not change significantly. Utilization of HA dropped from 52% to 39%, and RSA significantly increased from 11% to 28% (9). Investigating the causes of this shift in treatment trends is essential. At the same time, the standard of living has improved and patients have higher requirements for function after fracture, so it is necessary to choose the treatment modality with the best therapeutic effect by comparing different treatment modalities.

Although a network meta-analysis (10) has previously analyzed the comparative treatment efficacy of four of these five treatment modalities, the number of RCTs that could be included in the meta-analysis and the number of participants in the study have increased substantially from previous years given the recent updates in the data. Based on these we conducted this new network meta-analysis to re-compare the advantages and disadvantages between these treatment measures.

## Methods

### Study protocol and registration

Because all of the analyses were based on data from previously published studies, there was no need for ethical approval or patient consent. The Preferred Reporting Items for Systematic Reviews (PRISMA) and Meta-Analyses extension statement was used to generate this network meta-analysis (11). The *a priori* protocol for this NMA is available in the International Prospective Register of Systematic Reviews (PROSPERO): CRD42022341209.

### Search strategy

We searched PubMed, Embase, Cochrane Library for randomized controlled trials on proximal humeral fractures up to June 21, 2022. We have used the following search strategy in the Pubmed database: ((“Shoulder Fractures"[Mesh]) OR (((((((((((((Fracture, Shoulder[Title/Abstract]) OR (Fractures, Shoulder[Title/Abstract])) OR (Shoulder Fracture[Title/Abstract])) OR (Humeral Fractures, Proximal[Title/Abstract])) OR (Fracture, Proximal Humeral[Title/Abstract])) OR (Fractures, Proximal Humeral[Title/Abstract])) OR (Humeral Fracture, Proximal[Title/Abstract])) OR (Proximal Humeral Fracture[Title/Abstract])) OR (Proximal Humeral Fractures[Title/Abstract])) OR (Greater Tuberosity Fractures[Title/Abstract])) OR (Fracture, Greater Tuberosity[Title/Abstract])) OR (Fractures, Greater Tuberosity[Title/Abstract])) OR (Greater Tuberosity Fracture[Title/Abstract]))) AND (((((clinical[Title/Abstract]) AND (trial[Title/Abstract])) OR (clinical trials as topic[Mesh])) OR (clinical trial[Publication Type])) OR (random allocation[Mesh])).

### Study selection

The inclusion criteria: (1) all studies included in this NMA were randomized controlled trials (RCT); (2) participants in the study were on average over 70 years old and had a three- or four-part proximal humerus fracture; (3) two of the five therapy modalities (Conservative, locking plates, intramedullary nails, hemiarthroplasty, reverse total shoulder arthroplasty) are included in RCTs; (4) all contain follow-up results with a 12-month or longer follow-up period; (5) all of the literature featured is in English.

The exclusion criteria: (1) literature in a language other than English; (2) non-randomized controlled study; (3) patients in the study were diagnosed with a partial or two-part proximal humerus fracture; (4) other treatments for proximal humerus fractures.

### Data extraction and quality assessment

Data extraction and quality assessment from original articles was performed independently by two authors. For some papers for which we could not obtain the data we needed, we contacted the original authors to obtain the original data for evaluation. When our two independent writers disagree about how to process the retrieved data, we transfer it to a third author (senior head physician) to make a decision. For comparing the prognosis of treatment modalities, we use the Constant Score and reoperation rates. The extracted items included: (1) author, (2) time of publication; (3) study location; (4) treatment 1; (5) treatment 2; (6) sample size; (7) mean age; (8) sex; (9) fracture typing method; (10) fracture typing; (11) final available sample size; (12) follow-up time. Two independent reviewers utilized the Cochrane Risk of Bias Assessment tool to assess the quality of the included literature (12). The risk assessment is done through Review Manager 5.4 software. For each item was sored as high risk, uncertain risk or low risk. Similarly, for some risk assessment items that we judged to be of uncertain risk from the original article, we contacted the authors of the original article and redetermined them, following the opinion of the third author (senior chief physician) for all controversial judgments.

### Statistical analysis

For some of the literature data are missing and the relevant data are not available by contacting the original authors, we replaced and converted the missing data by the following methods: some studies did not report coefficients of variation such as standard deviations (SD), and we replaced them with the maximum standard deviation of the same rating scale used in other studies (13); if only the standard deviation of the baseline, difference is reported, then we calculate the standard deviation of the endpoint data using the correlation algorithm (14); we calculate the standard deviation for those who are only given a range by dividing the range difference by four (15).

For our Bayesian network meta-analysis, we used Stata software (Stata/MP 17.0. Revision 20 Apr 2021), Revman software (Version: 5.4.1), JAGS software, and the R software (Version 4.1.3), BlandAltmanLeh package, gemtc package, and riags package. We performed sampling simulations and calculations based on random effects model using MCMC method, plotted convergent diagnostic results as convergent diagnostic plots, trajectory plots and density plots, derived relative comparison results between various treatment measures, plotted league tables, and further calculated relative ranking and surface under the cumulative ranking curve (SUCRA) values. Means under the random effects model and fixed effects model were calculated and tested for homogeneity in the literature using the BlandAltmanLeh package; if all points are within 95% LoA, this indicates good homogeneity. The node-splitting approach was used to conduct consistency tests, and between direct and indirect comparisons, *p*-values greater than 0.05 were deemed to be favorable (16). If heterogeneity was found, further heterogeneity was tested, with *I*^2^ > 50% indicating heterogeneity, and for the overall results, we also looked at the total *I*^2^.pair and *I*^2^.cons, with values closer to 0 indicating no heterogeneity. We also performed sensitivity analyses: one literature was excluded at a time, and the remaining studies were combined for analysis to see if the results of the analysis would change.

## Results

### Research selection and characteristics of included study

**Figure 1:**
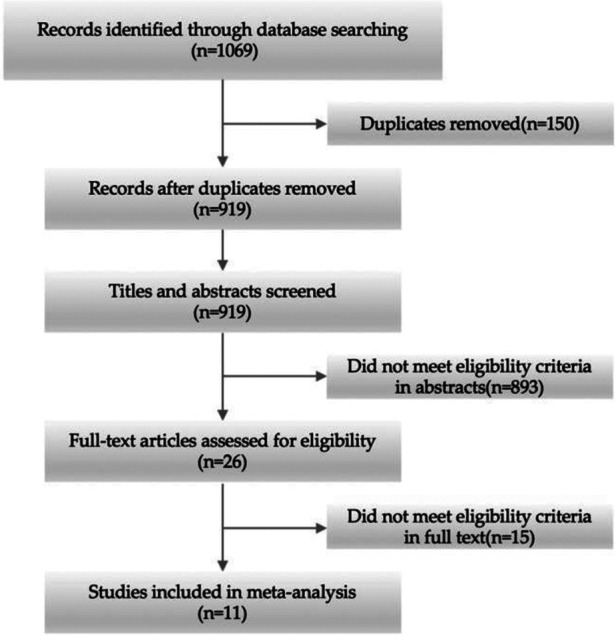
Flowchart of the trail selection.

After screening the literature and using our pre-defined search strategy, 11 studies with 648 participants were finally included in the analysis (17–27) (Figure 1). Three comparisons between HA and RSA, two each between Conservative and LP and Conservative and HA, and one each between RSA and LP, RSA and Conservative, HA and LP, and LP and IMN are included among the studies featured. There were no statistical differences in sample size, mean age, gender share, or fracture typing between the two groups before wise treatment, and all of the included literature met our pre-set screening criteria, were published after 2011, and all included two of the five treatment modalities for comparison (Table 1). The results of the other randomized controlled trials were based on the longest possible follow-up time retrieved, with the exception of one trial (20). We chose a 12-month follow-up period for this study because six patients were missed during the 12-to-24-month follow-up period, which we believe influenced the results significantly.

**Table 1:** Characteristics of data extracted from the included studies.

Study	Location	Treatment	Sample size	Mean age (year)	Sex (women, %)	Fracture typing method	Fracture type	Final available	Follow-up (months)
Boons et al., 2012	Netherlands	HA	25	76.4	96	Neer	4-part (100%)	24	12
		Conservative	25	79.9	92			23	
Boyer et al., 2021	France	IMN	49	74.7	70	Neer	3-part (84%) 4-part (16%)	43	66
		LP	50	75.0	68		3-part (69%) 4-part (31%)	42	
Cai et al., 2012	China	HA	19	71.1	86	Neer	4-part (100%)	15	24
		LP	13	72.4	88			12	
Fjalestad et al., 2012	Norway	LP	25	72.2	80	OTA/AO	3-part (52%) 4-part (48%)	23	12
		Conservative	25	73.1	96		3-part (52%) 4-part (48%)	25	
Jonsson et al., 2021	Sweden	RSA	48	80.5	95	Neer	3-part (49%) 4-part (51%)	41	28.8
		HA	51	78.6	86		3-part (56%) 4-part (44%)	43	
Lopiz et al., 2019	Spain	RSA	30	82.0	86	Neer	3-part (13%) 4-part (87%)	29	12
		Conservative	32	85.0	87		3-part (17%) 4-part (83%)	30	
Lass et al., 2020	Dutch	RSA	17	74.8	52	OTA/AO	3- or 4-part	14	12
		HA	14	75.0	64			11	
Olerud-a et al., 2011	Sweden	LP	30	72.9	80	Neer	3-part (100%)	27	24
		Conservative	29	74.9	83			28	
Olerud-b et al., 2011	Sweden	HA	27	75.8	85	Neer	4-part (100%)	24	24
		Conservative	28	77.5	86			25	
Fraser et al., 2020	Norway	RSA	64	75.7	92	OTA/AO	3-part (40%) 4-part (60%)	57	24
		LP	60	74.7	87		3-part (48%) 4-part (52%)	47	
Sebastia et al., 2014	Spain	RSA	31	74.7	87	Neer	3-part (16%) 4-part (84%)	31	28.5
		HA	30	73.3	83		3-part (13%) 4-part (87%)	29	

### Assessment of risk of bias

**Figure 2:**
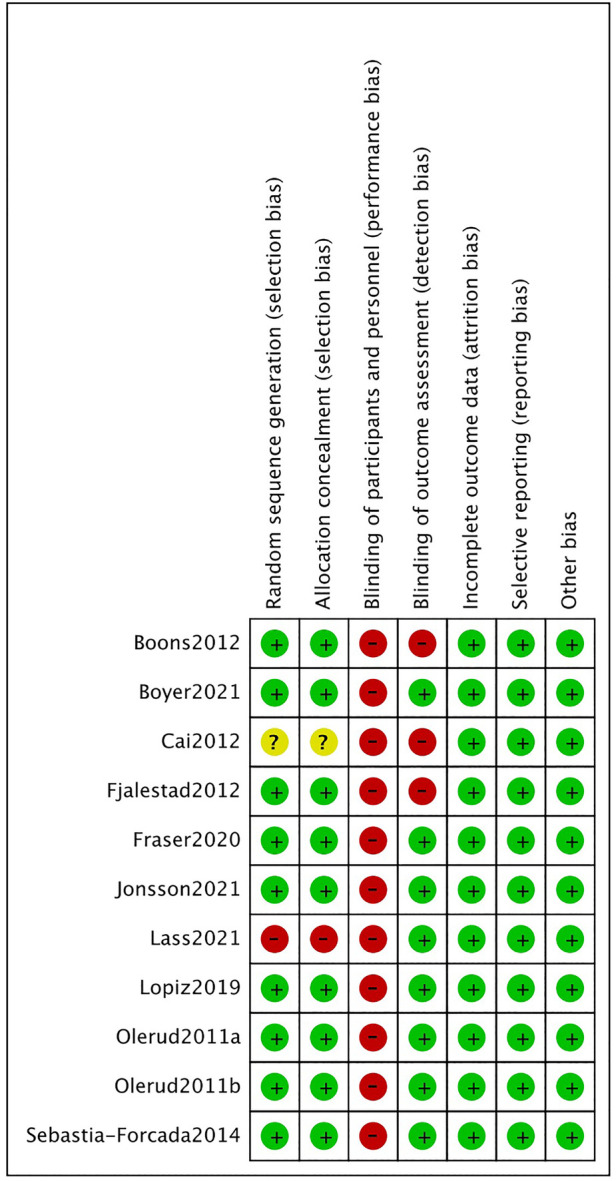
Risk of bias summary.

The outcome of the risk of bias assessment is shown in the Figure 2. For random sequence generation and allocation concealment, nine of the eleven studies were deemed low risk. Because of the small sample size, one study used “the method of minimization for allocation,” and while this method is recommended for clinical trials with small sample sizes and statistically indicates a small difference between the two groups, we ultimately rated it as high risk. The method of generating and assigning secret random number series in the original publication was not determined in another study. Due to the specific nature of surgical treatment, all subjects inevitably know the surgical procedure they underwent, and therefore we judged all trials to be high risk in terms of allocation concealment. Nine studies reported the assessment of outcome indicators by independent investigators on the outcome blinded assessment, two of which (25, 26) were assessed by independent investigators on the outcome indicators at follow-up only at 24 months after surgery, and our extracted data were also at 24 months, so the risk assessment was different this time from the results of a previous meta-analysis, which we evaluated as low risk. All studies were evaluated as low risk in terms of incomplete outcome bias, selective reporting bias, and other bias items.

### Statistical analysis

**Figure 3:**
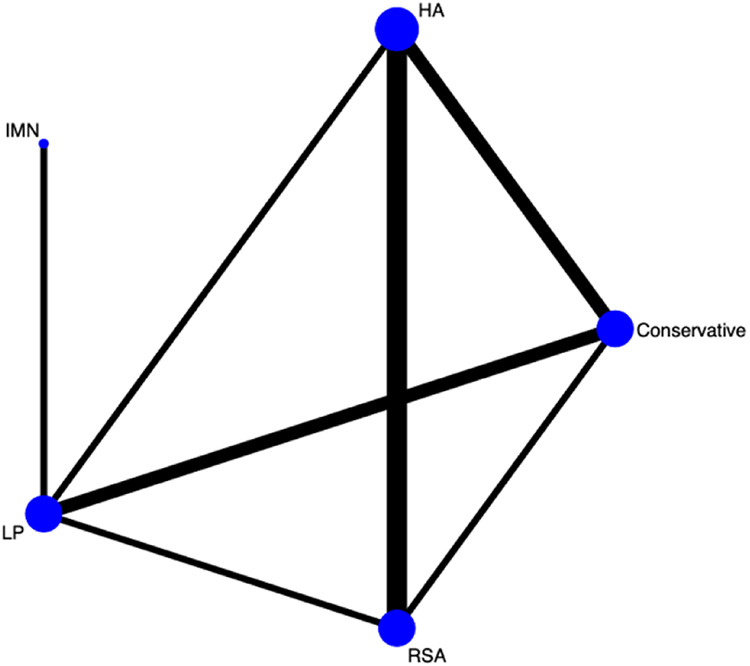
Network of the comparisons for the Bayesian network meta-analysis.

**Figure 4:**
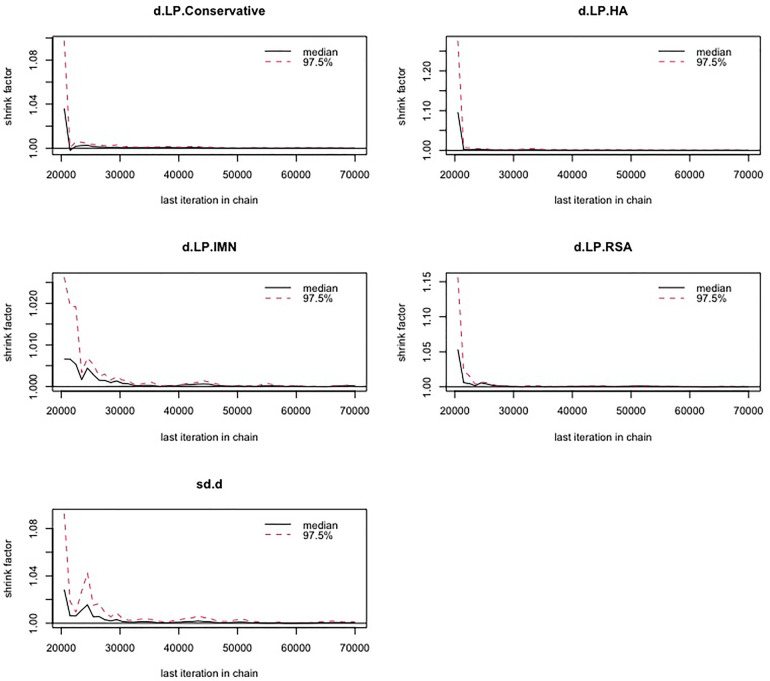
Diagnostic plot.

**Figure 5:**
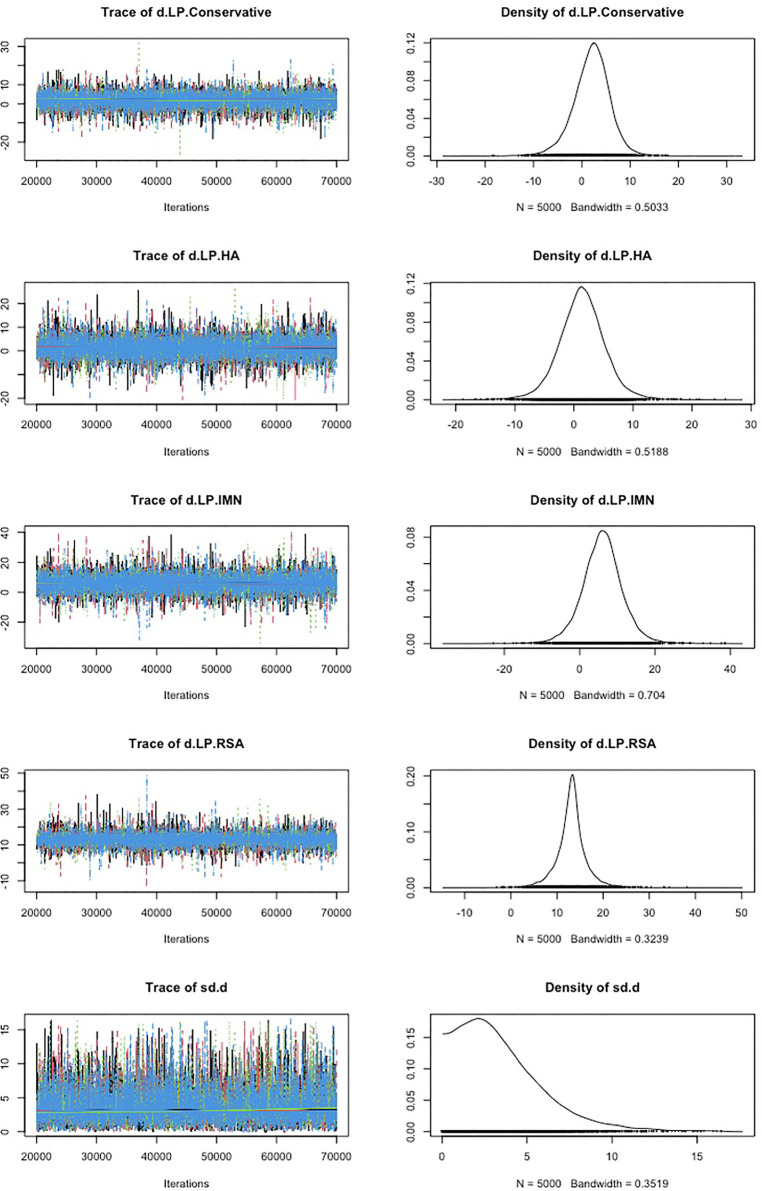
Trace, and density plot.

**Figure 6:**
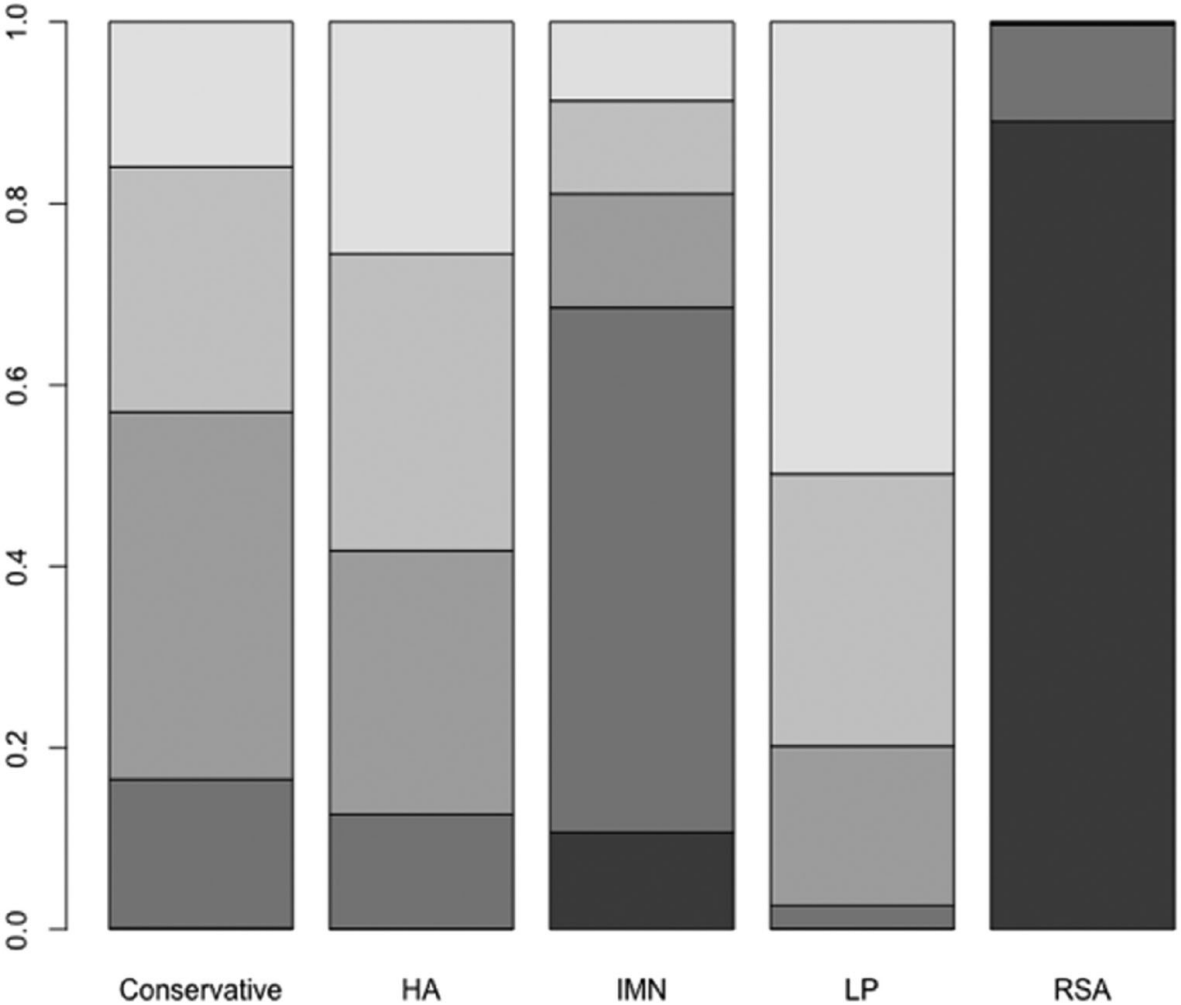
Rank probability of constant score.

**Figure 7:**
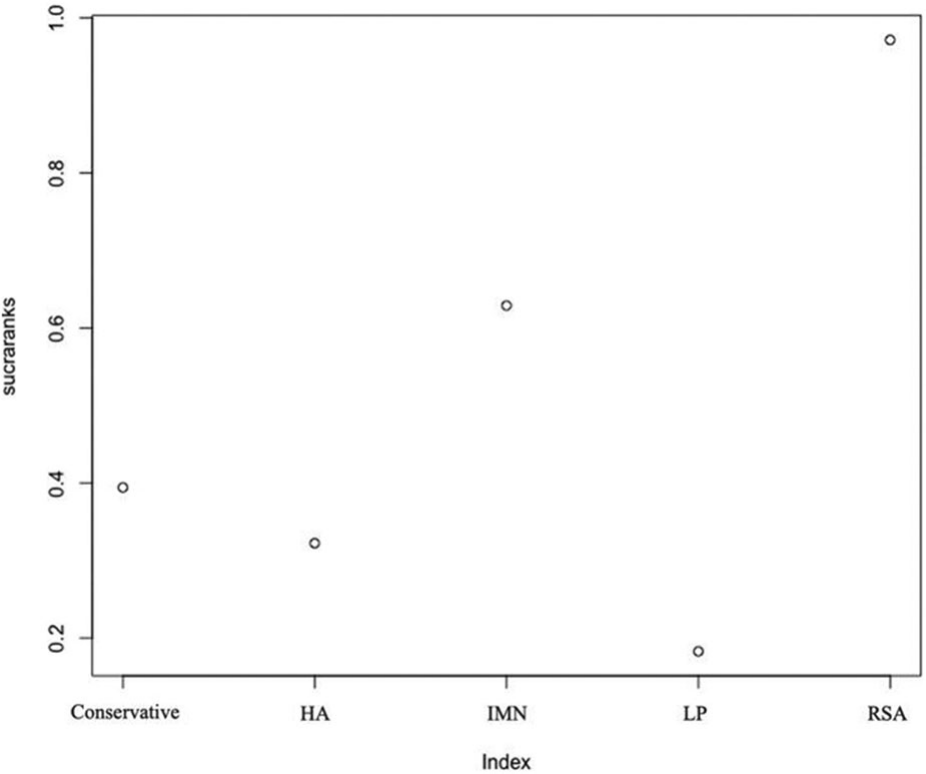
SUCRA values of constant score.

**Figure 8:**
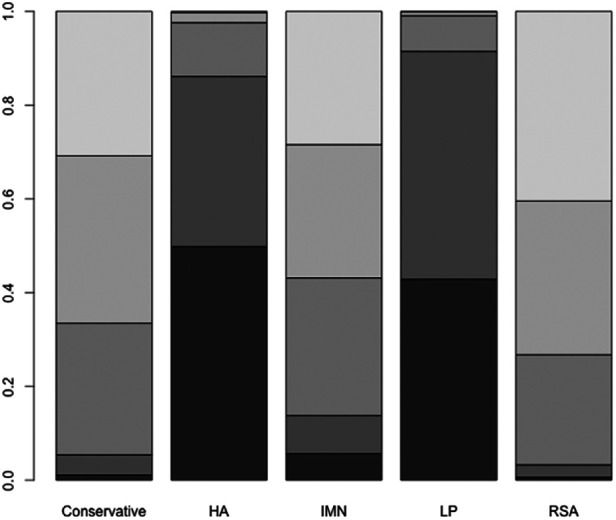
Rank probability of reoperation.

**Figure 9:**
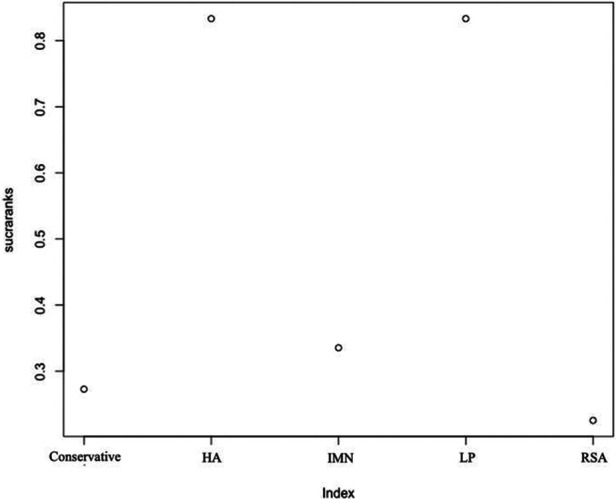
SUCRA values of reoperation.

First, we used Stata software to generate a network evidence map (Figure 3). Through R software, we performed sampling simulations and calculations using the MCMC method based on a random effects model that fits well and has heterogeneity equal to 0 (ratio 0.9279, *I*^2 ^= 0%). Additionally, we acquired the convergence diagnostic data, and we generated the diagnostic plot (Figure 4), trace, and density plot based on these findings (Figure 5). The potential scale reduction factor (PSRF) is calculated (Table 2), and the model is a satisfactorily converged model in combination with the convergence diagnostic plots and PSRF values. The league table between the various interventions is shown in Table 3. The comparison between various interventions yielded ranking results between species treatment measures, and combining the ranking results (Figure 6) with the SUCRA values (Figure 7), we can obtain: the RSA ranked first (0.9716625), the IMN ranked second (0.6289875), the Conservative ranked third (0.3942625), the HA ranked fourth (0.3222875), LP ranked fifth (0.1828000). We also calculated the reoperation ranking (Figure 8) and SUCRA values (Figure 9): LP (0.8333125), HA (0.8333000), IMN (0.3352875), Conservative (0.2726875), RSA (0.2254125).

**Table 2:** Potential scale reduction factor (PSRF).

	Point est.	Upper C.I.
d. LP. Conservative	1	1
d. LP. HA	1	1
d. LP. IMN	1	1
d. LP. RSA	1	1
sd. d.	1	1

**Table 3:** The league table between the various interventions.

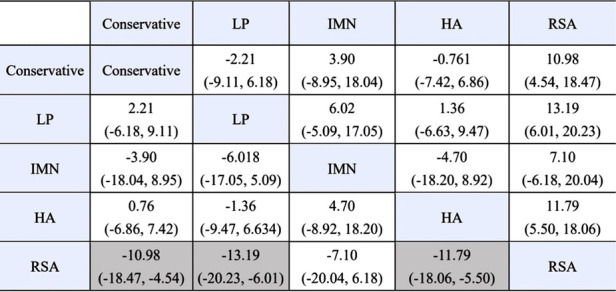

**Figure 10:**
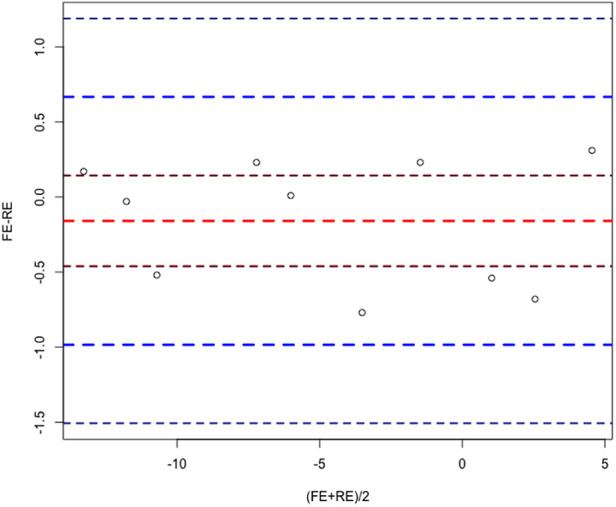
Bland–Altman diagram.

We calculated the means under the random effects model and the fixed effects model, and implemented the Bland-Altman method based on the BlandAltmanLeh package of R software to compare the results of the random effects model and the fixed effects model, and the results are shown in the Figure 10, and all points are within 95% LoA, which indicates great homogeneity.

**Figure 11:**
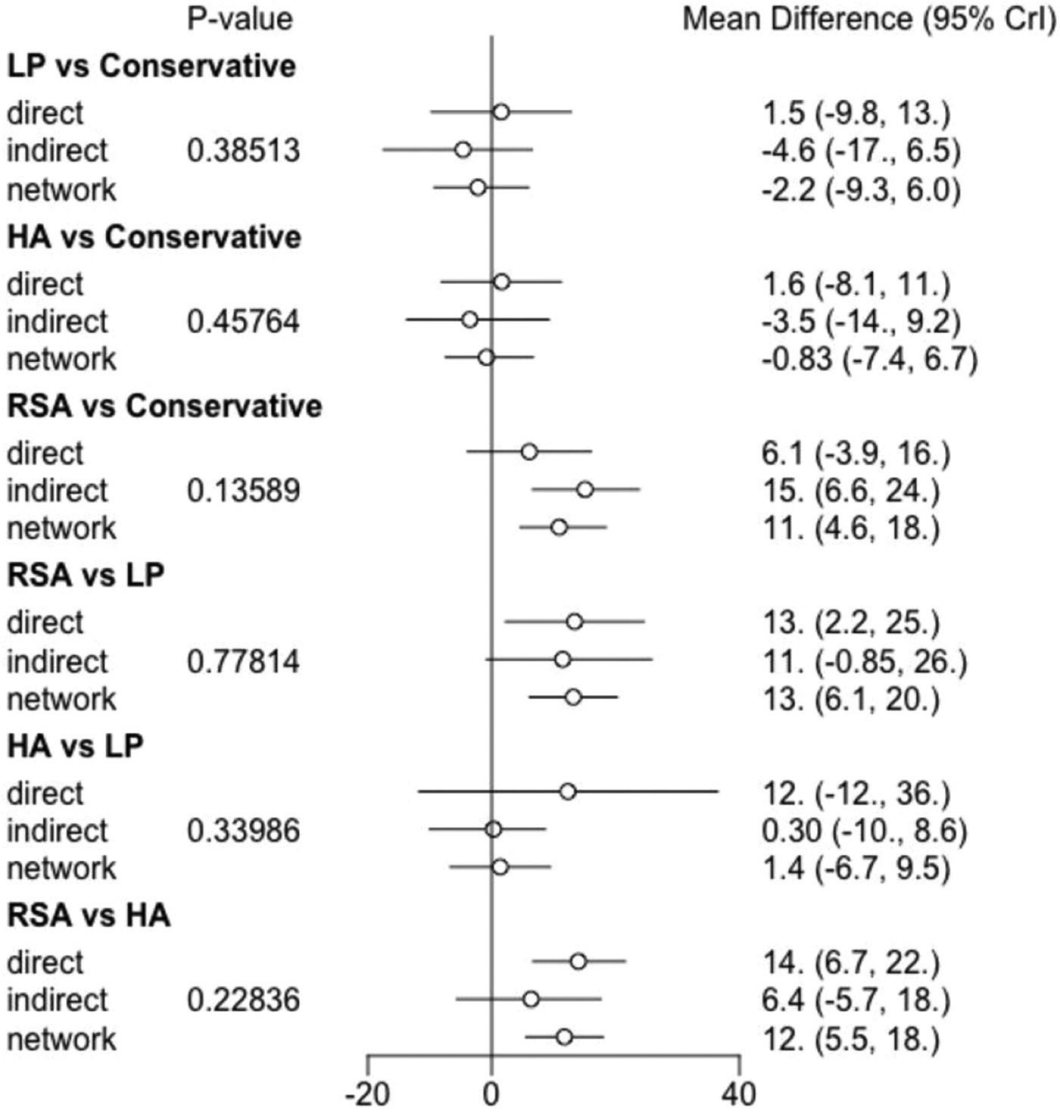
Inconsistency plot of this network meta-analysis (constant score).

**Figure 12:**
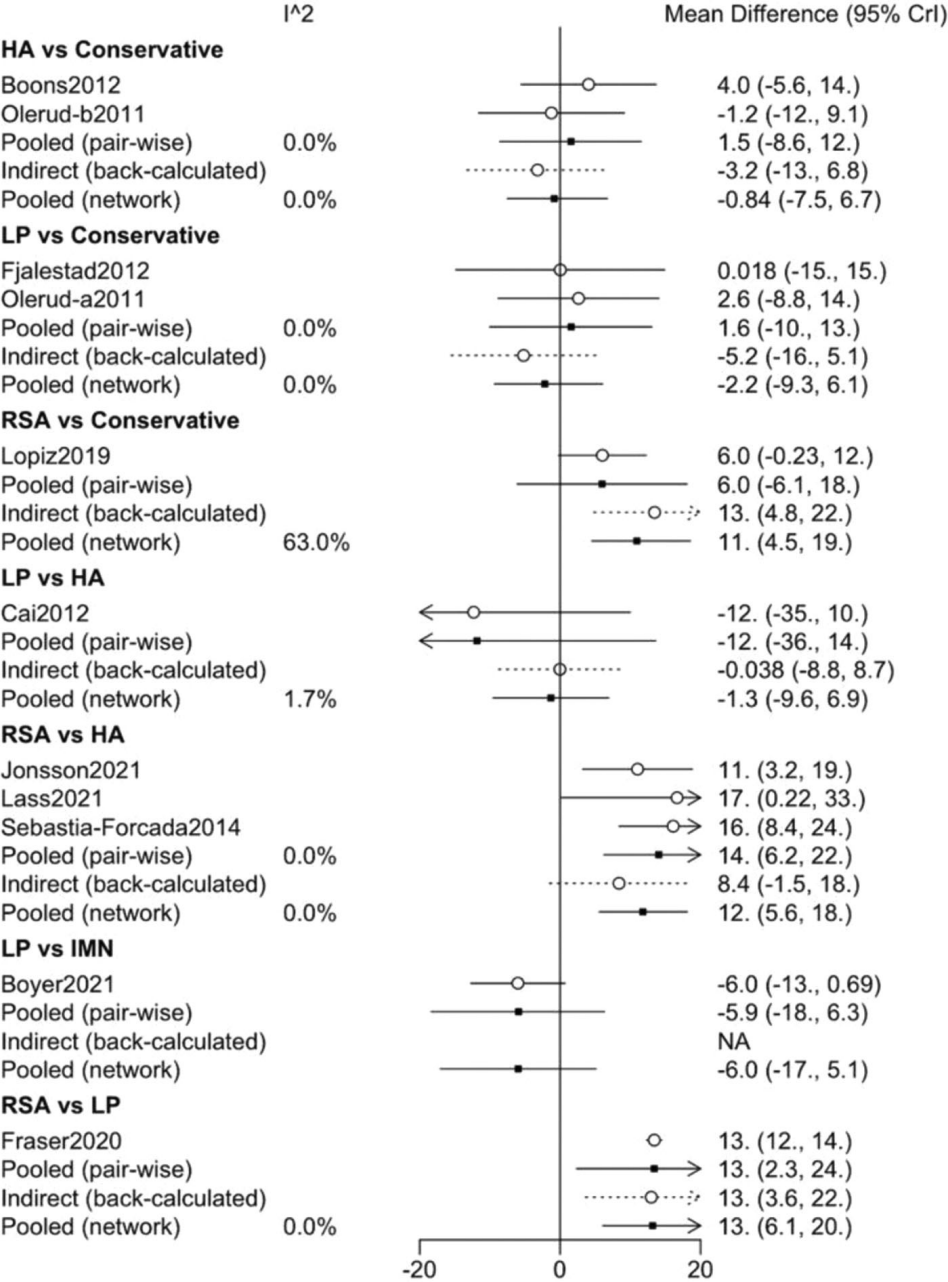
Heterogeneity plot of this network meta-analysis (constant score).

We performed a consistency test using the nodal splitting method, and the results are shown in the forest plot (Figure 11), and the *p*-values for both direct and indirect comparisons of the various treatment measures were greater than 0.05, indicating that the results of direct and indirect comparisons of the interventions were consistent and had good consistency. I In addition, we also performed a heterogeneity check, as shown in the Figure 12, with the exception of heterogeneity between RSA and Conservative (*I*^2^ > 50%). Conservative, which had heterogeneity (*I*^2^ > 50%). The total *I*^2^.pair and *I*^2^.cons are zero, so there is no significant heterogeneity in the present data from the overall results. After sequentially excluding the literature and re-running the meta-analysis, none of the results of the analysis were changed from the previous ones.

## Discussion

The best therapy for three- or four-part proximal humerus fractures is still up for debate. The absence of high-quality randomized controlled trials with large sample sizes is currently the most pressing problem in selecting the optimal treatment based on evidence. And completing such a trial would need not just multicenter teamwork, lengthy hours, and a significant sum of money, but also a number of practical and ethical concerns (it is difficult to randomize the choice of surgical approach in clinical practice). The existing studies were compiled and analyzed to draw comparative stage conclusions by conducting a meta-analysis of existing randomized controlled trials. On the other hand, by combining the analysis of the shortcomings also provide directions for future research, targeted clinical trials also improve the current predicament to a certain extent.

This is the first Bayesian network meta-analysis of 3 or 4 component fractures of the proximal humerus after Du (10). We redefined the open reduction and internal fixation, and for the first time incorporated IMN in the analysis, unlike Du. The results of our analysis were to some extent identical to the results of Du's net meta-analysis: both rated RSA as having the best clinical effect and LP as having the worst clinical effect. However, unlike previous results, our analysis showed slightly higher ranking results for Conservative than HA on the constant score, while having a low reoperation rate. And our newly added comparator IMN came in second in constant score and third in reoperation rate, but there is only one study on IMN. Studies assessing the effectiveness of IMN in 2- or 3-part proximal humeral fractures have been conducted. In a randomized controlled trial of IMN and LP for two-part proximal humerus fractures, Zhu (28) found no significant differences in clinical outcomes between the two treatment options and a lower complication rate for IMN at 3-year follow-up; however, Gracitelli's (29) study found that IMN and LP produced similar clinical and radiographic outcomes but had a higher complication and reoperation rate for two- or three-part fractures. More randomized controlled trials are therefore required to confirm the effectiveness and prevalence of unfavorable events with reference to IMN.

We can observe from the league tables that none of the comparisons were statistically significant, with the exception of Conservative and RSA, LP and RSA, and HA and RSA. Combining these results, we cannot conclude that Conservative has a superior clinical outcome than HA, despite the fact that Conservative was somewhat higher in the combined ranking this time.

Retrospective study analysis of German Health Insurance Fund data by Stolberg-Stolberg et al. (30) supported our analysis by demonstrating that, after being tailored to the patient's risk profile, reverse shoulder arthroplasty resulted in statistically significantly lower mortality and fewer major adverse events in long-term outcomes. However, the findings of Köppe's (31) study, which also examined this foundation database, were unexpected: there was a rise in in-hospital major adverse events and in-hospital perioperative complications with RSA in comparison to locking plate fixation, even after potential confounding factors like age, gender, and risk status were taken into account. Since few studies have compared in-hospital adverse events among treatment modalities, it is prudent to use RSA for proximal humeral fractures if the risk of in-hospital complications is noticeably higher, even though RSA has significant advantages over LP in terms of reoperation rates and other medium- to long-term evaluation criteria. In this area, more study is required.

To our surprise, our study also produced approximate SUCRA values for the HA and LP reoperation rates, which were different from many earlier findings. We reanalyzed the included literature by excluding each piece one at a time, and after excluding Fraser's (21) literature, we discovered that the reoperation rate SUCRA values for LP were significantly higher than HA. This finding may be related to the fact that, despite being consistently categorized as locking plate internal fixation, the technique of the procedure and the plates have been evolving over time (32–34). This implies that the results of earlier human research need to be viewed with greater objectivity because improved surgical procedures and plates may have reduced the incidence of negative events like reoperation rates for LP-treated proximal humeral fractures.

There is another issue that cannot be ignored: cost effectiveness. The ProFHER (PROximal Fracture of the Humerus: Evaluation by Randomisation) trial demonstrated that non-surgical treatment for PHF did not produce better results than surgical treatment in terms of patient outcomes. According to a base case economic analysis for the UK region, the cost of surgical interventions within 2 years was, on average, £1780.73 more expensive per patient than the cost of non-surgical interventions (35). Therefore, non-surgical treatment may be a more suitable treatment option in developing nations or economically underdeveloped regions. RSA has been demonstrated to be the most cost-effective method for treating complex PHF in elderly patients. The incremental cost per quality-adjusted life year (QALY) obtained with RSA relative to HA was C$13,679 (36). The anticipated cost discrepancy for QALY is $16.8 per 100 persons treated, whereas the cost differential for RSA against HA translates to a savings of $99,626 per 100 people treated (37). Additionally, RTSA has a 66% likelihood of being the most cost-effective treatment choice at a willingness-to-pay threshold of C$50,000/QALY (38). The results of the analysis of cost-effectiveness also explain, to some extent, the changes in treatment trends.

This network meta-analysis, however, has a number of drawbacks. (1) Despite the increase in the number of trials and participants included in our meta-analysis compared with the previous one, there were no more than two comparisons between all treatment modalities except between RSA and HA, which limited the significance of our conclusions to some extent. (2) The different percentages of three- and four-part fractures in different trials, the reliability of the fracture classification, the technical variability of the surgeons in different studies (32), the subjective variability of constant score scores and the lack of uniform standards for postoperative functional exercise may have influenced the judgment of outcome indicators to some extent. (3) Due to the small number of included trials and the differences in reporting indicators of outcome among different trials, we only selected constant score and reoperation rate as outcome indicators for analysis, which also had some limitations. In contrast to the results of our analysis, Köppe's (31) study showed an increase in major adverse events and surgical complications with RSA compared to locking plate fixation after controlling for potential confounding variables such as age, gender, and risk status. Despite the fact that this is a retrospective study, it does highlight the limits of using reoperation rates to reflect adverse outcomes. (4) The follow-up duration for eight of the eleven studies that were included was no longer than 2 years. The short follow-up period of the included trials was also a drawback.

## Conclusion

In conclusion, from our present meta-analysis, it can be concluded that the best treatment for proximal humeral 3- or 4-part fractures is RSA, there is no statistically significant difference in constant score scores between other treatment measures, but the ranking is IMN, Conservative, HA, LP. the reoperation rate is ranked from highest to lowest LP, HA, IMN, Conservative, RSA.

## Data Availability

The original contributions presented in the study are included in the article/Supplementary Material, further inquiries can be directed to the corresponding author/s.
